# Integrating multi-omics data reveals IL-8 positive cancer-associated fibroblasts as mediators of chemotherapy-induced tumor progression in breast cancer

**DOI:** 10.3389/fimmu.2026.1878482

**Published:** 2026-07-20

**Authors:** Huifeng Liao, Huayan Li, Jin Song, Junhua Dong, Xue Bai

**Affiliations:** 1 Department of General Surgery, The First Medical Center of Chinese People’s Liberation Army General Hospital, Beijing, China; 2 The Second School of Clinical Medicine, Southern Medical University, Guangzhou, China; 3 Department of General Surgery, The Seventh Medical Center of Chinese People’s Liberation Army General Hospital, Beijing, China; 4 Department of Breast Surgery, Fengtai Maternal and Child Health Hospital, Beijing, China

**Keywords:** cancer-associated fibroblasts, IL-8, Mendelian randomization, patient-derived tumor-like cell clusters, tumor microenvironment

## Abstract

**Background:**

Recent studies have shown that while chemotherapy kills tumor cells, it may also induce adaptive changes in cells within the tumor microenvironment, particularly cancer-associated fibroblasts (CAFs), which could paradoxically promote tumor progression. This study aimed to investigate the role of CAFs exposed to paclitaxel (PTX) or doxorubicin (DOX) in tumor progression and explore the underlying mechanisms.

**Methods:**

We examined the effects of PTX- or DOX-exposed CAFs on tumor migration and invasion through *in vitro* experiments. Bioinformatics analyses including Mendelian randomization, BayesPrism, and scPagwas were performed to elucidate the clinical relevance of IL-8 + CAFs in breast cancer. Additionally, *in vitro* experiments were conducted to confirm the tumor-promoting effects of CAFs-derived IL-8 and explore the underlying molecular mechanisms.

**Results:**

Our findings showed that CAFs exposed to PTX or DOX further promoted tumor migration and invasion compared to untreated CAFs. Notably, PTX and DOX significantly increased IL-8 expression levels in CAFs, suggesting that IL-8 may be a key cytokine mediating the tumor-promoting effect of CAFs exposed to chemotherapeutic agents. Interestingly, this tumor-promoting effect could be partly reversed by reparixin, an IL-8 receptor inhibitor. Mendelian randomization analysis confirmed that both IL-8 eQTL and plasma IL-8 levels were significantly associated with breast cancer risk. Further insights from BayesPrism and scPagwas indicated that IL-8 + CAFs were associated with clinical prognosis and may play a critical role in breast cancer progression. Furthermore, we confirmed the tumor-promoting effect of CAFs-derived IL-8. Mechanistically, IL-8 may modulate apoptosis by activating the NF-κB pathway, thereby inhibiting tumor cell apoptosis and leading to chemoresistance. Clinically, high IL-8 expression correlated with chemoresistance and poor survival in breast cancer patients.

**Conclusion:**

Our study suggests that the upregulation of IL-8 in CAFs following PTX or DOX treatment may contribute to chemotherapy-mediated tumor progression. These findings provide potential avenues for improving chemotherapy outcomes.

## 1 Introduction

Chemotherapy plays an instrumental role in the pre-operative, post-operative, and palliative treatment of breast cancer (BRCA), but the recurrence and metastasis caused by chemoresistance remain major obstacles preventing patients from achieving a durable remission and eventual cure. Most of the previous studies on chemoresistance and metastasis have focused on the genetic alterations that occur within tumor cells, but in recent years, chemotherapy-induced chemoresistance and metastasis have gradually emerged as a new research hotspot (1–4). Despite the effectiveness of chemotherapy as a first-line cancer treatment, accumulating evidence from animal models indicates that while chemotherapy exerts antitumor effects, it also promotes several off-target effects that induce tumor metastasis (5–7). Usually, chemotherapy aims to destroy tumor cells alone, but the systemic administration of cytotoxic drugs inevitably affects non-tumor cells, thus promoting the host response (8). The host response to chemotherapy is a result of the systemic release of multiple cytokines and the mobilization of various host cells, including macrophages, endothelial cells, and fibroblasts, which influence the response to therapeutics and the overall disease outcome to some extent (9–13). As cancer research continues to advance, more and more studies have reported that chemotherapy promotes cellular and molecular responses in host cells which promote chemoresistance and tumor progression.

Generally, the undesired side effects of chemotherapy on the tumor microenvironment (TME) are the major factors that induce metastasis (14, 15). Indeed, there is a dynamic interaction between tumor cells and non-tumor cells in the TME during chemotherapy, which allows tumor cells to escape from the primary tumor site to the peripheral blood, thereby promoting chemoresistance and metastasis (16–18). For example, DNA damage caused by DOX induces thymic endothelial cells to release IL-6 and tissue inhibitor of metallopeptidase 1, which protects tumor cells from the genotoxic effects of chemotherapy (19). PTX induces the infiltration of tumor-associated macrophages into the tumor, which then release cathepsin proteases, thereby preventing tumor cells from chemotherapy-induced cell death (20). Similarly, platinum drugs induce the activation of bone marrow mesenchymal stem cells, causing them to secrete unique fatty acids that lead to the development of chemoresistance (21). In conclusion, chemotherapy induces various cells in the TME to secrete cytokines through different pathways, which protects the tumor cells from the cytotoxic effects of chemotherapeutic agents and then promotes tumor progression. CAFs are one of the most important cell subpopulations in the TME, however, only a few studies have focused on the effects of CAFs exposed to chemotherapeutic agents on tumor cells. Therefore, it is clinically relevant to reveal the potential mechanism of CAFs in chemotherapy-mediated tumor progression.

Neoadjuvant chemotherapy (NAC) provides long-term benefits for the clinical treatment of BRCA patients. However, in addition to direct effects on tumor cells, the administration of cytotoxic drugs alters the phenotype and characteristics of non-tumor cells, especially CAFs, thus promoting therapy resistance and metastasis. Our study highlights the important function of CAFs in chemotherapy-induced progression and proposes that chemotherapy combined with targeted IL-8 may be an effective treatment strategy for inhibiting tumor progression.

## 2 Materials and methods

### 2.1 Human serum and tissue samples

We collected serum samples from 50 patients during NAC treatment, and tumor tissue from 100 patients pre- and post-NAC. All patients were diagnosed with primary invasive ductal carcinoma of the breast by core needle biopsy. Inclusion criteria were: (1) histologically confirmed primary BRCA; (2) received PTX− or DOX−based NAC; (3) available pre− and post−treatment tissue samples. Exclusion criteria were: (1) prior history of other malignancies; (2) receipt of radiotherapy or targeted therapy before NAC; (3) distant metastasis at initial diagnosis. Hormone receptor (ER/PR) and HER-2 status were determined by IHC. Molecular subtypes were classified according to the St. Gallen criteria. The sensitivity of patients to chemotherapy was determined according to the Response Evaluation Criteria in Solid Tumors (RECIST) guidelines.

### 2.2 Primary fibroblast isolation and culture

Primary CAFs were isolated from the tumor tissue, while normal fibroblasts (NFs) were isolated from normal breast tissue. The fresh tissue samples were minced into pieces, centrifuged to remove the supernatant, and were added to the digestion solution (0.01 mg/ml collagenase II + 0.01 mg/ml hyaluronidase) for 15 min. The homogenate was collected and passed through a 100 μm sieve and centrifuged (1500 rpm, 5 min) to remove the supernatant. The cells were then resuspended in DMEM/F12 media and placed in an incubator (37 °C, 5% CO_2_). We seeded 1×10^6^ of CAFs in a 6-well plate and allowed them to grow up to 80%. The conditioned medium (CM) was collected from serum-free media after culturing for 24 or 48 h and was stored at -80 °C for further use after centrifugation.

### 2.3 Cell culture and transfection

MCF-7 cells were cultured in DMEM medium with 10% fetal bovine serum (FBS), and MDA-MB-231 cells (Boster, Wuhan, China) were cultured in L-15 media with 10% FBS. The cell lines were authenticated through STR analyses and mycoplasma tests were performed each month through qPCR. Patient-derived tumor-like cell clusters (PTCs) were isolated from BRCA tissues and cultured in a low-attachment-surface petri-dish with advanced DMEM (22). According to the manufacturer’s protocol, CAFs were transfected with the IL-8-pcDNA3.1 (Sangon, Shanghai, China) using Lipofectamine 3000 (Invitrogen, USA). Tumor cells were transfected with Bcl-2 or Bcl-xL siRNA (Genepharma, China) using Lipofectamine 3000 (Invitrogen, USA). The transfection efficiency was shown in **Supplementary Figures 2A, B**.

### 2.4 Data acquisition and processing

To investigate the causal association of IL-8 with BRCA risk, we performed Mendelian randomization (MR) analysis. The exposure data for genetically predicted IL-8 expression levels (eQTL) were obtained from the GWAS database (ukb-a-213), and the circulating IL-8 protein levels were sourced from the GWAS Catalog (GCST90274817). The survival information for BRCA was obtained from the TCGA database. The GSE23399 and GSE268662 datasets were downloaded from the GEO database. GSE23399 was used to study the expression of IL-8 in CAFs before and after chemotherapy. Single-cell RNA-sequencing data from 5 BRCA patients (GSE268662) were analyzed using the Seurat package (v 4.3) to comprehensively characterize the CAFs signature. Quality control was performed to filter out low-quality cells. Specifically, cells expressing fewer than 200 genes or more than 10,000 genes were excluded. Additionally, cells with a mitochondrial gene percentage greater than 20% were removed to eliminate dying cells. After filtering, a total of 14,825 cells and 25,603 genes were retained for subsequent normalization and dimensionality reduction. CAFs were annotated with 4 marker genes, including *Vimentin*, *FAP*, *PDGFRα*, and *PDGFRβ*.

### 2.5 BayesPrism deconvolution analysis and scPagwas analysis

BayesPrism (23) was employed to compute the score for each cell subpopulation by adding the deconvolution scores integrals of each cell state for each subpopulation. Utilizing BayesPrism, we performed deconvolution on the bulk RNA-seq data from TCGA-BRCA to investigate the correlation between IL-8 + CAFs and clinical outcomes. Based on the annotations of different cell types from scRNA-seq, scPagwas (24) was used to calculate the Trait Related Score (TRS) for each cell type to evaluate their potential role in BRCA.

### 2.6 Cell viability assay

The Cell Counting Kit-8 (CCK-8) assay was used to measure the viability of tumor cells. The tumor cells were seeded in a 96-well culture plate and co-cultured with CAFs CM for 48 hours. For the drug sensitivity assay, the tumor cells were treated with PTX or DOX for 48 hours after co-culture with CAFs CM for 48 hours. 10 μL of CCK-8 solution (Boster, Wuhan, China) was added to each well for 1 hour and then the absorbance was measured at 450 nm with a microplate reader. The area of ​​all PTCs clusters in the wells was measured to assess the drug effect. The clusters were photographed on days 0 and 7, and only those with diameters greater than 40 μm at both time points were selected for area calculation. The formula for calculating PTCs cell viability after chemotherapy was performed as previously described (22).

### 2.7 Wound healing, invasion, and colony formation assays

Wound healing, invasion, and colony formation assays were performed as previously described (25).

### 2.8 Enzyme-linked immunosorbent assay and quantitative real-time PCR

The IL-8 concentration in human serum samples and CAFs CM was quantified by the ELISA kit (Boster, Wuhan, China). The mRNA levels of IL-8 were quantified by qRT-PCR.

### 2.9 TUNEL formation assay

The TUNEL Apoptosis Assay Kit (Beyotime, Shanghai, China) was used to detect apoptosis in the tumor cells and xenograft tissues. Five areas were randomly selected under the fluorescence microscope and the percentage of apoptotic cells was calculated.

### 2.10 Western blotting

Cells were lysed with RIPA buffer. Following SDS-PAGE, transfer, and blocking, the polyvinylidene difluoride membranes were incubated with the respective primary antibodies overnight at 4 °C. The membranes were washed and incubated with the secondary antibodies. Enhanced chemiluminescence reagent was used to detect the protein bands. Protein levels were normalized to GAPDH levels.

### 2.11 Immunohistochemistry

Immunohistochemistry (IHC) staining was performed to detect the expression of IL-8, α-smooth muscle actin (α-SMA), Bcl-2, Bcl-xL, and Bax in human and mouse tumor tissues, according to standard protocols. The IL-8, α-SMA, Bcl-2, and Bcl-xL were purchased from CST, USA. The Bax and p-p65 were purchased from Beyotime, Shanghai, China. For IL-8 staining evaluation, cells with more than 5% positivity were considered positive, while those with less than 5% were considered negative. For evaluation of α-SMA, Bcl-2, Bcl-xL, and Bax staining, the percentage of positive cells was calculated as follows: <5% = 0; 5%-25% = 1; 25%-50% = 2; 50%-75% = 3; and >75% = 4. There were four levels of staining intensity: 0, negative; 1, weak; 2, medium; and 3, strong. Multiplying the two scores resulted in the following combined score: 0, negative; 1-4, weak expression; 5-8, moderate expression; and 9-12, strong expression.

### 2.12 H&E staining

The paraffin sections were baked at 60 °C until the wax melted, and washed with xylene, ethanol, and running water for dewaxing. Hematoxylin staining was performed for 10 min, followed by differentiation for 20 s, blueing for 5 min, and eosin staining for 3 min, with running water washes between steps. Finally, the sections were dehydrated and sealed with neutral gum seal.

### 2.13 Animal experiments

16 four-week-old female NSG nude mice were purchased from the Medical Discovery Leader (MDL, Beijing, China). They were randomly divided into four groups and injected with tumor cells (1 × 10^7^) mixed with Matrigel subcutaneously into the left armpit. Compared with the control group, the experimental group was injected with IL-8 (100 ng/ml) continuously for 5 days, beginning on the 12th day. Tumor diameter was measured every 3 days and tumor volume (mm^3^) was estimated by volume = (shortest diameter) (mm)^2^ × (longest diameter) (mm) × 0.5.

### 2.14 Kaplan-Meier plotter database

Kaplan-Meier Plotter was used for survival analysis including relapse-free survival (RFS) and distant metastasis-free survival (DMFS) in BRCA patients (26). “Auto select best cutoff” and “only JetSet best probe set” were chosen in the analysis.

### 2.15 Statistical analysis

All continuous variables were subjected to normality testing using the Shapiro-Wilk (n < 50) or Kolmogorov-Smirnov (n ≥ 50) test prior to analysis. The associations between the protein levels and various clinicopathological parameters were analyzed with Pearson’s χ^2^ test. Quantitative data were compared between the control and the treatment groups by analysis of variance. Homogeneity of variance was assessed by Levene’s test; when *P* > 0.05, LSD *post-hoc* test was used for pairwise comparisons, and when *P* ≤ 0.05, Dunnett’s T3 *post-hoc* test was applied. Statistical analyses were performed using SPSS 24.0 software. For single-cell differential expression analysis, *P* values were adjusted using the Benjamini-Hochberg method to control the False Discovery Rate (FDR). A threshold of FDR < 0.05 was considered statistically significant. In MR analysis, the IVW method was used for causal estimation, supported by MR-Egger and Cochran’s Q tests to assess pleiotropy.

## 3 Results

### 3.1 Chemotherapy-pretreated CAFs exhibit superior pro-tumorigenic capabilities compared to CAFs

**Figure 1:**
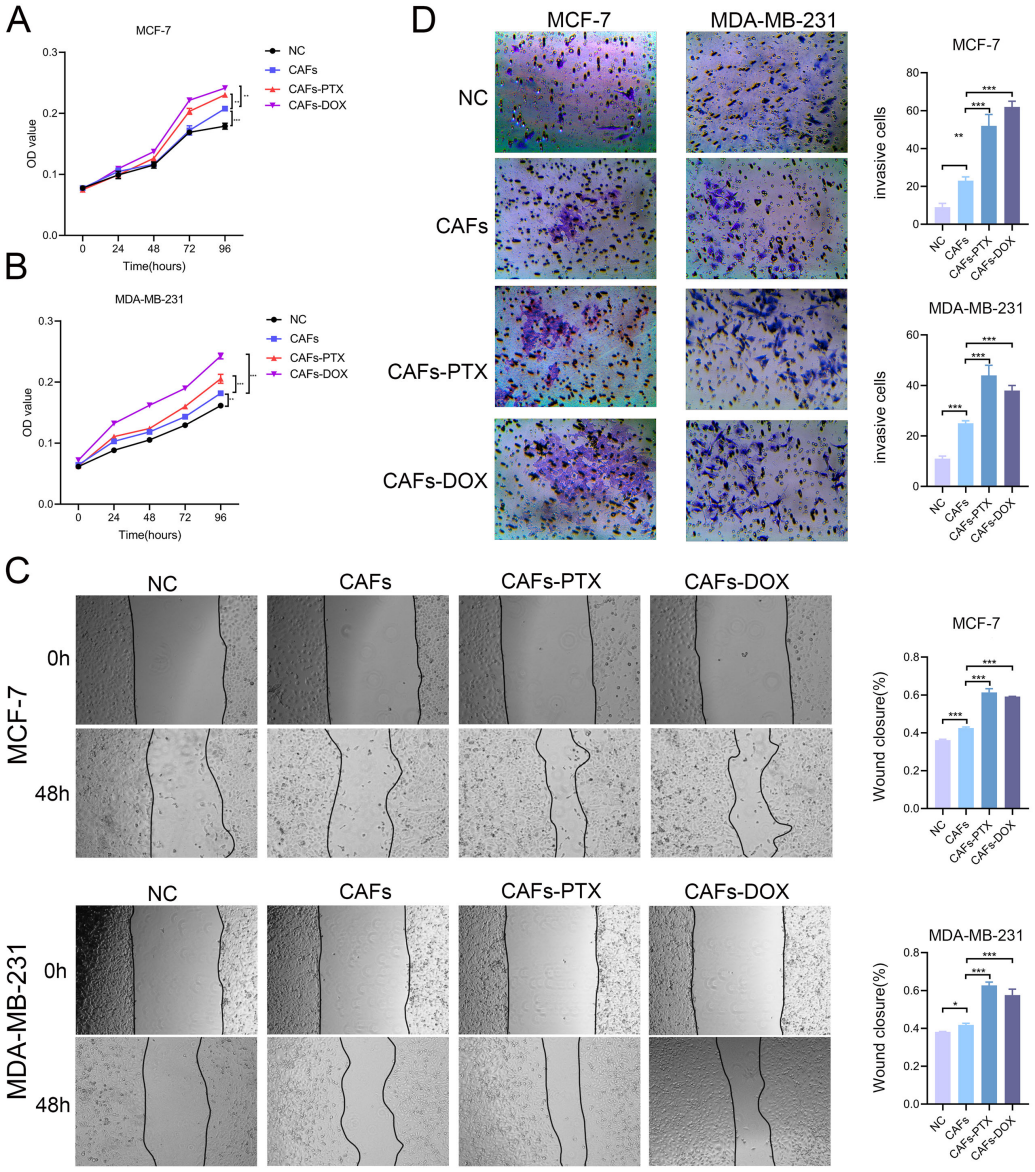
Effects of CAFs exposed to chemotherapeutic agents on tumor cells. **(A-D)** By using CCK-8 assay **(A, B)**, wound healing assay **(C)**, and invasion assay **(D)**, we detected the proliferation, wound healing, and invasion abilities of MCF-7 and MDA-MB-231 cells that co-culture with CM from CAFs or CAFs treated with chemotherapeutic agents. The data were expressed as mean ± SD for at least triplicate experiments. *P < 0.05, **P < 0.01, ***P < 0.001.

CAFs in the TME may undergo adaptive responses during chemotherapy, which subsequently lead to tumor progression. To investigate the effect of chemotherapy-pretreated CAFs on tumor cells, we exposed CAFs to PTX (0.01 μM) or DOX (0.01 μM) for 24 hours, then replaced the medium with serum-free culture medium for 24 hours before collecting the CM. As expected, CM from CAFs promoted the proliferation, wound healing, and invasion of MCF-7 and MDA-MB-231 cells. It was worth noting that CAFs treated with PTX or DOX further promoted the proliferation, wound healing, and invasion of tumor cells compared to CAFs (**Figures 1A–D**).

### 3.2 Chemotherapy promoted IL-8 expression *in vitro* and *in vivo*

**Figure 2:**
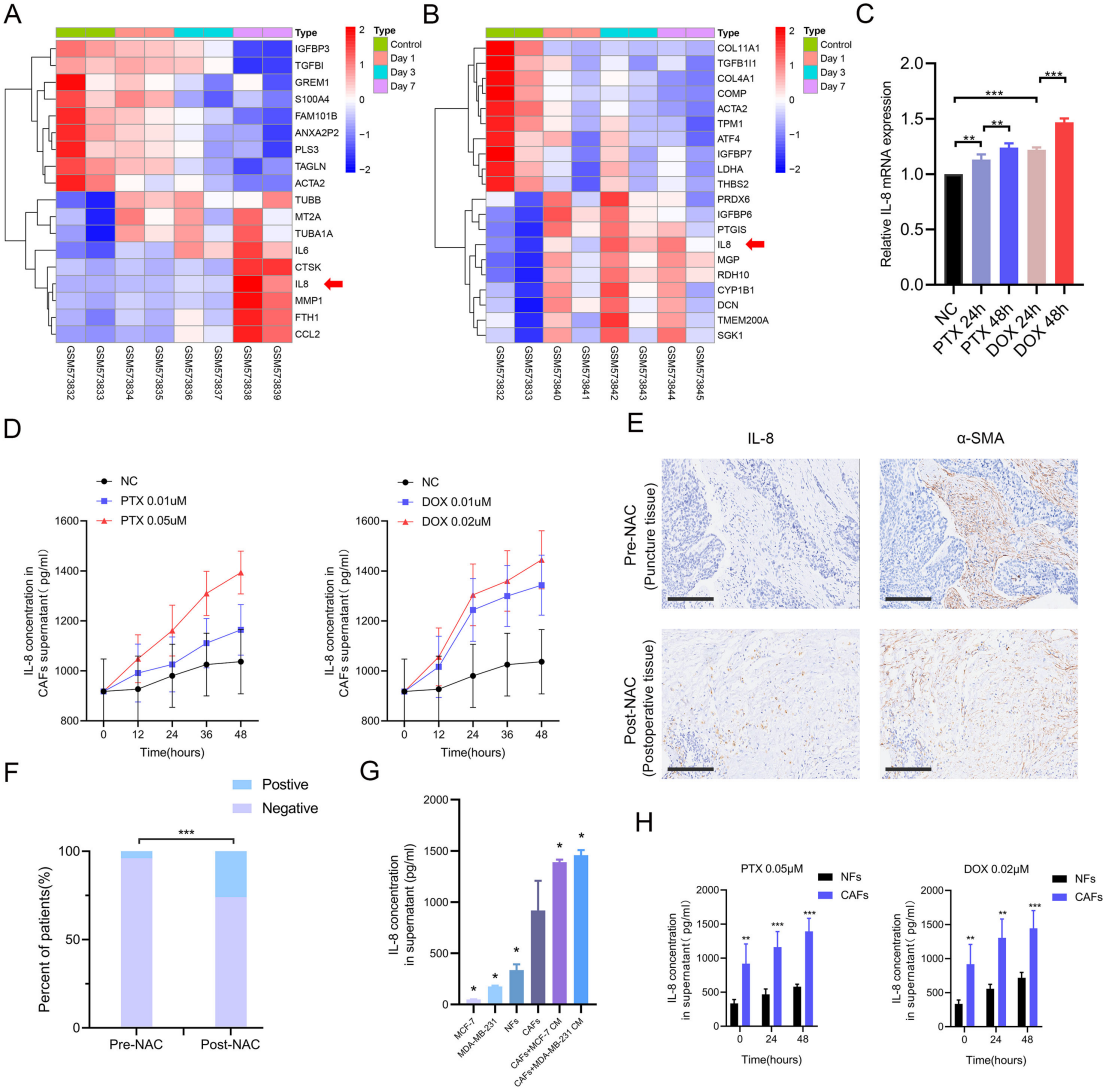
PTX and DOX promoted IL-8 expression in CAFs *in vitro* and *in vivo*. **(A, B)** The IL-8 gene expression change in CAFs after being treated with PTX (Day 3 vs NC, P < 0.05) or DOX in Days 1, 3, and 7 (Day1, 3, and 7 vs NC, P < 0.05). **(C, D)** IL-8 expression in CAFs increased in a dose- and time-dependent manner detected by qRT-PCR **(C)** and ELISA assay **(D)**. **(E)** IL-8 expression was elevated in the tumor tissues post-NAC and IL-8 was mostly localized in fibroblasts expressing α-SMA. Scale bar, 200 µm. **(F)** IHC assessment of IL-8 expression in the tumor stroma in BRCA patients (n=100) pre- and post-NAC. Patients were classified into 2 categories according to IL-8 staining in the tumor stroma: positive and negative. **(G)** The IL-8 concentration in supernatant of breast tumor cells and fibroblasts. **(H)** IL-8 concentration in CAFs CM was notably higher than corresponding NFs. The data were expressed as mean ± SD for at least triplicate experiments. *P < 0.05, **P < 0.01, ***P < 0.001.

To analyze the underlying mechanisms of this phenomenon, we downloaded the GSE23399 dataset from the GEO database. GSE23399 was used to analyze the gene expression changes of CAFs following PTX or DOX treatment. Interestingly, both PTX and DOX treatment upregulated IL-8 expression in CAFs (**Figures 2A, B**). We then used qPCR and ELISA assay to examine the expression of IL-8 in CAFs before and after chemotherapy. We found that PTX and DOX significantly promoted IL-8 expression in CAFs, and IL-8 levels in the CM rose in a dose- and time-dependent manner (**Figures 2C, D**). To further verify whether chemotherapy promoted IL-8 expression *in vivo*, we performed IHC on samples from BRCA patients who received PTX- or DOX-based NAC. IHC staining showed that IL-8 expression level was significantly upregulated in breast tumor stroma post-NAC in comparison to the pre-NAC samples. Moreover, IL-8 was mostly located in the CAFs expressing α-SMA (**Figures 2E, F**). The above data indicated that chemotherapy promoted IL-8 expression from the CAFs both *in vitro* and *in vivo*. Additionally, IL-8 levels in CAFs were significantly higher than in tumor cells and NFs, and after co-culturing with tumor cells, the expression of IL-8 in CAFs CM was significantly increased (**Figure 2G**). Interestingly, chemotherapy also promoted IL-8 expression in NFs, but the IL-8 concentration in supernatant from NFs was considerably lower than that from CAFs (**Figure 2H**).

### 3.3 IL-8 involved in the enhanced tumor-promoting effect of chemotherapy-pretreated CAFs

**Figure 3:**
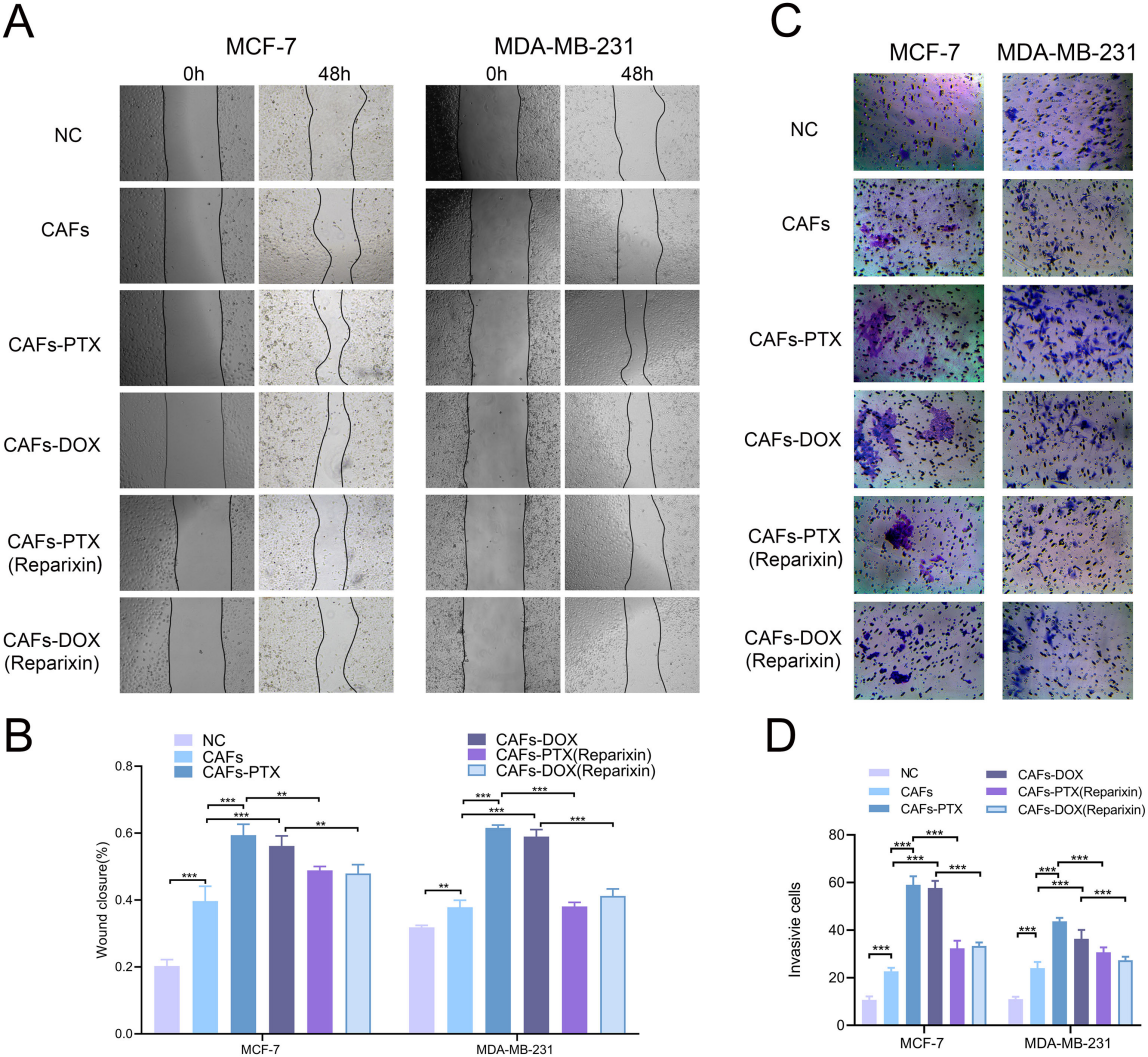
IL-8 involved in the enhanced tumor-promoting effect of chemotherapy-pretreated CAFs. **(A-D)** The tumor-promoting effect of CAFs that exposed to PTX or DOX was partly reversed by reparixin. The enhanced migration and invasion of tumor cells induced by chemotherapy-pretreated CAFs were effectively attenuated by reparixin. **: *P* < 0.01 , ***: *P* < 0.001.

To determine whether IL-8 was a key cytokine involved in chemotherapy-induced tumor progression, we used 100 nM Reparixin (IL-8 receptor inhibitor) to treat tumor cells for 24 hours and then co-cultured with CAFs. We found that Reparixin partially reversed the tumor-promoting effect of CAFs that were treated with chemotherapeutic agents (**Figures 3A–D**). These findings suggest that IL-8 may play a key role in mediating the tumor-promoting effects of chemotherapy-pretreated CAFs.

### 3.4 Mendelian randomization reveals a causal relationship between IL-8 and BRCA

**Figure 4:**
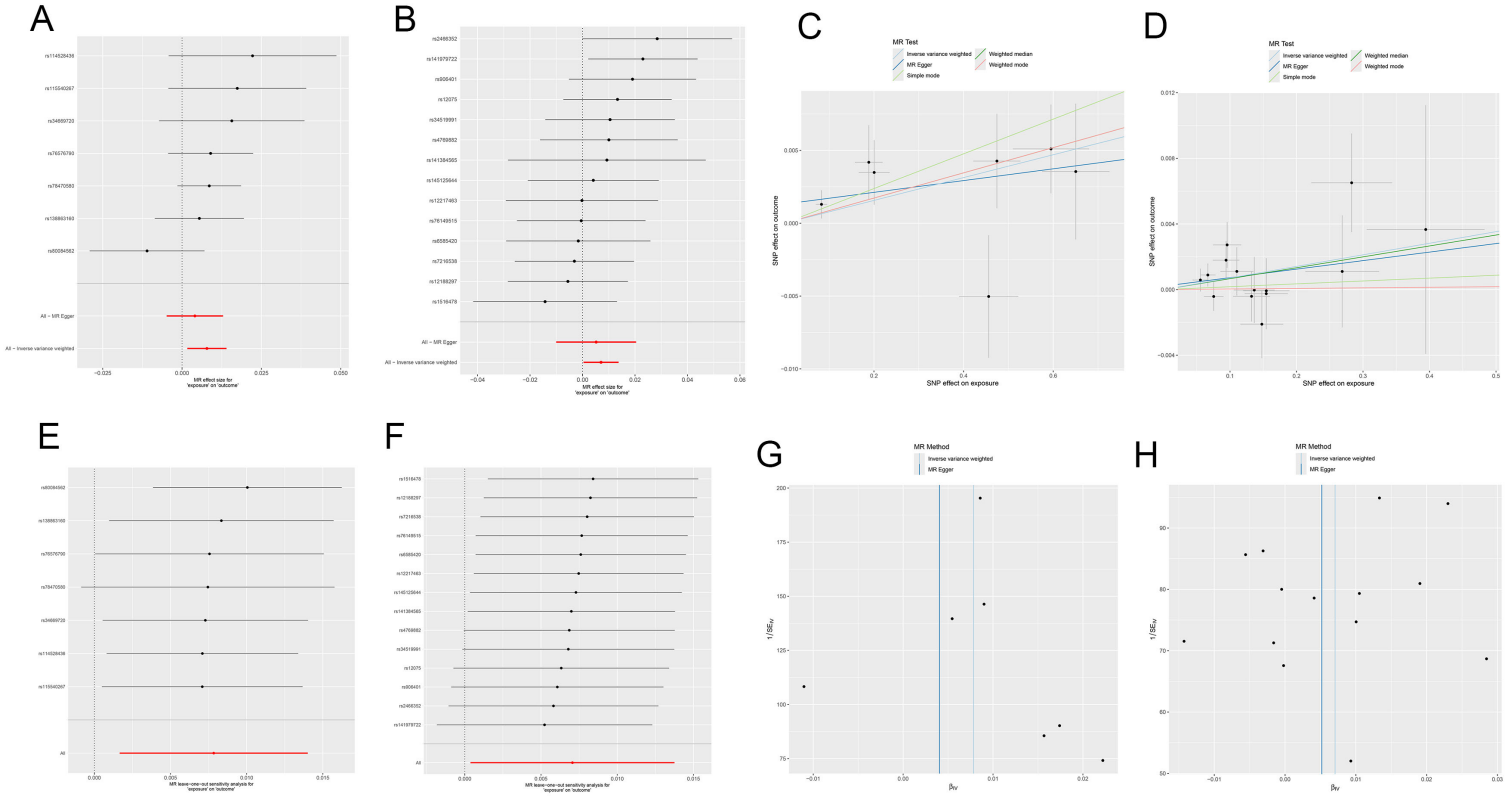
MR analyses of IL-8 and BRCA. **(A, B)** Causal effect of IL-8 eQTL **(A)** and plasma IL-8 **(B)** on BRCA. **(C, D)** Scatter plot depicting the causal relationships between the IL-8 eQTL **(C)** and plasma IL-8 **(D)** with BRCA. **(E, F)** Leave-one-out analyses of IL-8 in BRCA. **(G, H)** Funnel plot of MR analyses.

We used MR analysis to further analyze the causal relationship between IL-8 and BRCA. The results showed that both IL-8 eQTL (**Figures 4A, C**) and plasma IL-8 levels (**Figures 4B, D**) were positively correlated with the risk of BRCA. Leave-one-out analyses confirmed that no single SNP biased the causal inference (**Figures 4E, F**). Moreover, there was no heterogeneity or pleiotropy in the MR analyses (**Figures 4G, H**, **Tables 1**, **2**).

**Table 1:** Heterogeneity and pleiotropy of individual SNPs in IL-8 eQTL.

		Heterogeneity		Pleiotropy
		MR Egger	IVW	MR Egger
Exposure	Outcome	P	P	P
IL-8	Breast cancer	0.381	0.353	0.307

**Table 2:** Heterogeneity and pleiotropy of individual SNPs in the inflammatory factor IL-8.

		Heterogeneity		Pleiotropy
		MR Egger	IVW	MR Egger
Exposure	Outcome	P	P	P
IL-8	Breast cancer	0.522	0.598	0.794

### 3.5 The potential role of IL-8 + CAFs in BRCA

**Figure 5:**
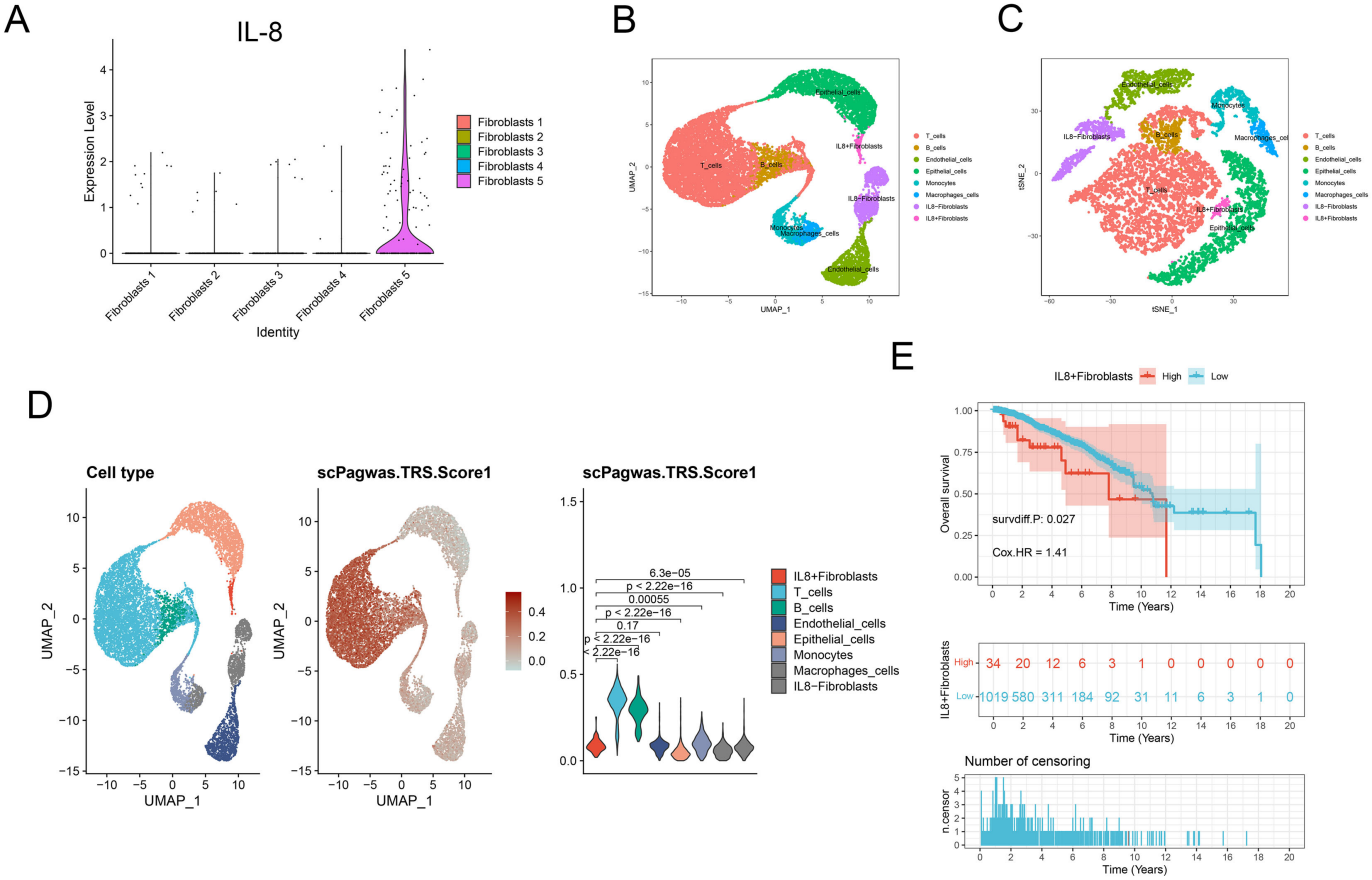
The potential role of IL-8 + CAFs in BRCA. **(A)** The expression of IL-8 in different fibroblast subtypes. **(B, C)** UMAP **(B)** and tSNE **(C)** visualization of different cell types. **(D)** TRS of different types of cells. **(E)** Survival differences between the high- and low- IL-8 + CAFs group.

We identified and characterized distinct subpopulations of CAFs in the GSE268662 dataset. The processing and quality control of the single-cell data were detailed in **Supplementary Figures 1A–E**. Notably, IL-8 was barely expressed in Fibroblasts 1-4, but highly expressed in Fibroblasts 5. Based on this differential expression, Fibroblasts 5 were defined as IL-8 + CAFs, whereas other Fibroblasts were defined as IL-8-CAFs (**Figures 5A–C**). By integrating the scRNA-seq and GWAS data using the scPagwas algorithm, we calculated the TRS for each cellular subpopulation. **Figure 5D** showed a significant correlation between IL-8 + CAFs and BRCA compared with IL-8 - CAFs (0.0871 vs 0.0731, *P* = 6.3e-05). IL-8 + CAFs may be the key subpopulation of BRCA. We then used BayesPrism to deconvolute the bulk RNA-seq data from TCGA-BRCA. BRCA patients were categorized into high- and low- IL-8 + CAFs groups according to the deconvolution scores. Survival analysis revealed that the high IL-8 + CAFs group was associated with a poorer prognosis (**Figure 5E**). Together, these insights suggest that IL-8 + CAFs potentially play a crucial role in the pathogenesis of BRCA.

### 3.6 Effects of CAFs-derived IL-8 on tumor cells

**Figure 6:**
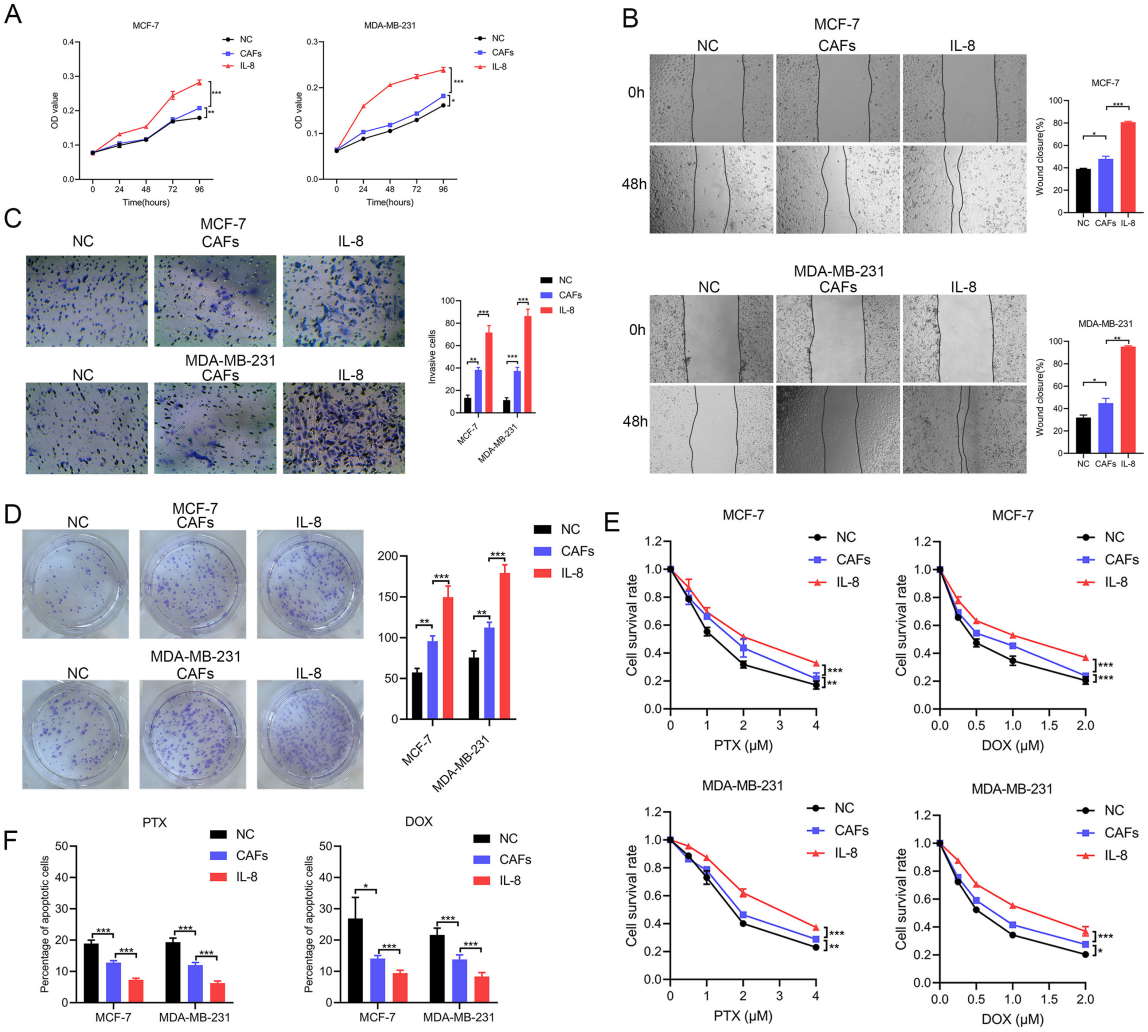
The tumor-promoting effects of CAFs-derived IL-8. **(A-C)** The proliferation **(A)**, wound healing **(B)**, and invasion **(C)** abilities of tumor cells. **(D)** IL-8 (100 ng/ml) promoted colony formation ability of tumor cells. **(E)** CAFs-derived IL-8 decreased the chemosensitivity of tumor cells to PTX and DOX. **(F)** TUNEL assay showed that CAFs-derived IL-8 reduced PTX- or DOX-induced apoptosis. *: *P* < 0.05, **: *P* < 0.01 , ***: *P* < 0.001.

**Figure 7:**
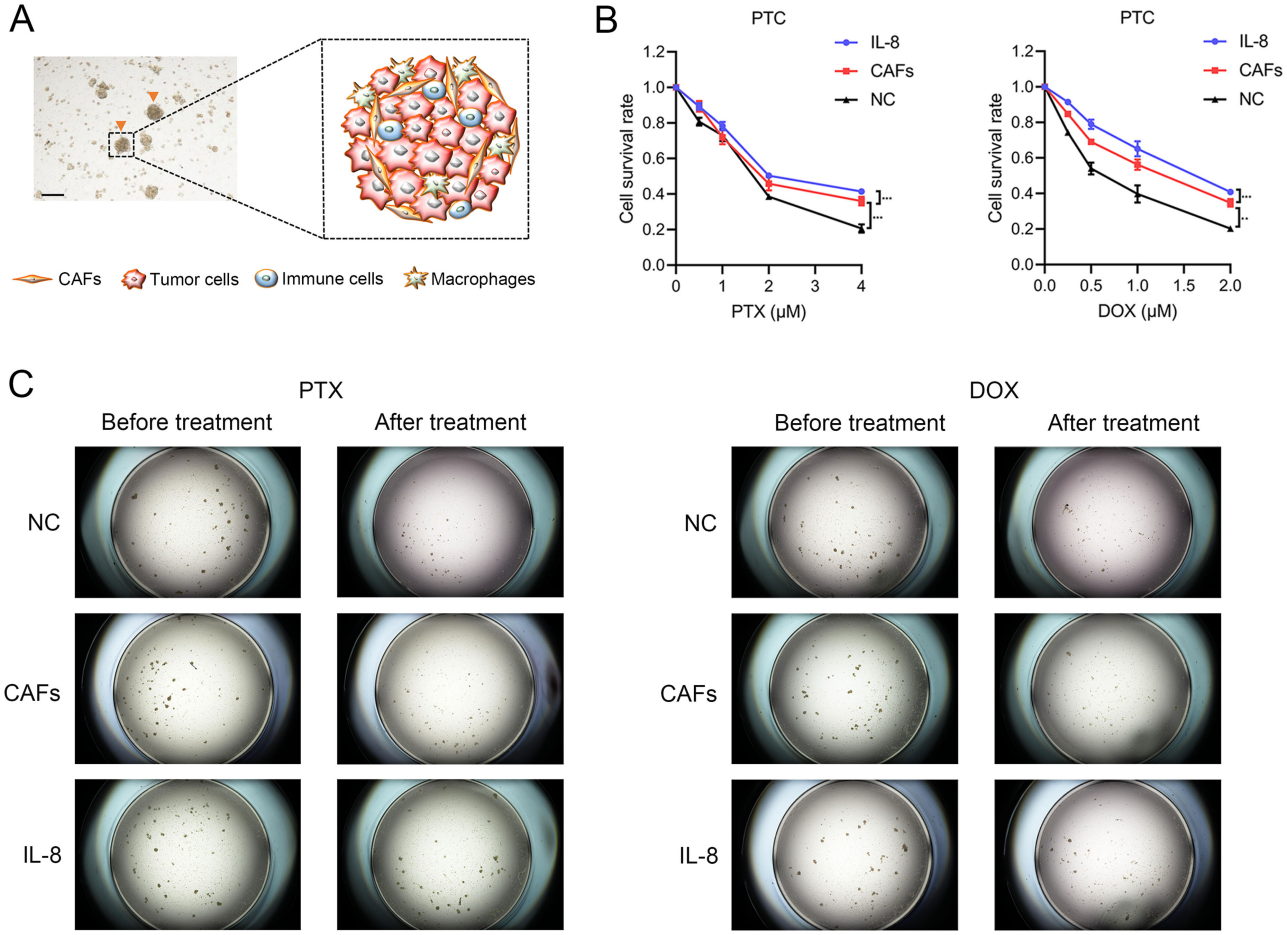
The effects of IL-8 on the chemosensitivity of PTCs. **(A)** Schematic diagram of PTCs, which contained primary tumor cells, fibroblasts, macrophages, and a variety of immune cells that highly recapitulated TME in terms of structure and function. Scale bar, 100 µm. **(B)** IL-8 enhanced the resistance of PTCs to PTX and DOX. **(C)** Area of PTCs in each well before and after treatment. Data were shown as means ± SD for three independent experiments. *P < 0.05, **P < 0.01, ***P < 0.001.

We transfected CAFs with IL-8 plasmid to detect the effects of IL-8 on tumor cells. The results showed that CAFs-derived IL-8 promoted the proliferation, wound healing, and invasion of MCF-7 and MDA-MB-231 cells (**Figures 6A–C**). The colony formation assay showed that recombinant IL-8 (100 ng/ml) significantly enhanced the colony formation ability of tumor cells (**Figure 6D**). Moreover, CAFs-derived IL-8 decreased the chemosensitivity of tumor cells to PTX or DOX (**Figure 6E**). Meanwhile, the TUNEL apoptosis assay showed that CAFs-derived IL-8 significantly reduced PTX- or DOX-induced tumor apoptosis (**Figure 6F**). To further elucidate the effect of IL-8 on chemosensitivity and more closely replicate the environment in the patient’s tumor tissue, we used 3D cultured micro-tumor PTCs for the experiment. PTCs are a novel primary tumor cell model, wherein the different cell types from the TME can self-assemble and proliferate, thereby replicating the heterogeneous breast TME in terms of structure and function (22). The results showed that CAFs-derived IL-8 enhanced the chemoresistance of PTCs (**Figures 7A–C**).

### 3.7 IL-8 promoted the growth of tumor xenografts

**Figure 8:**
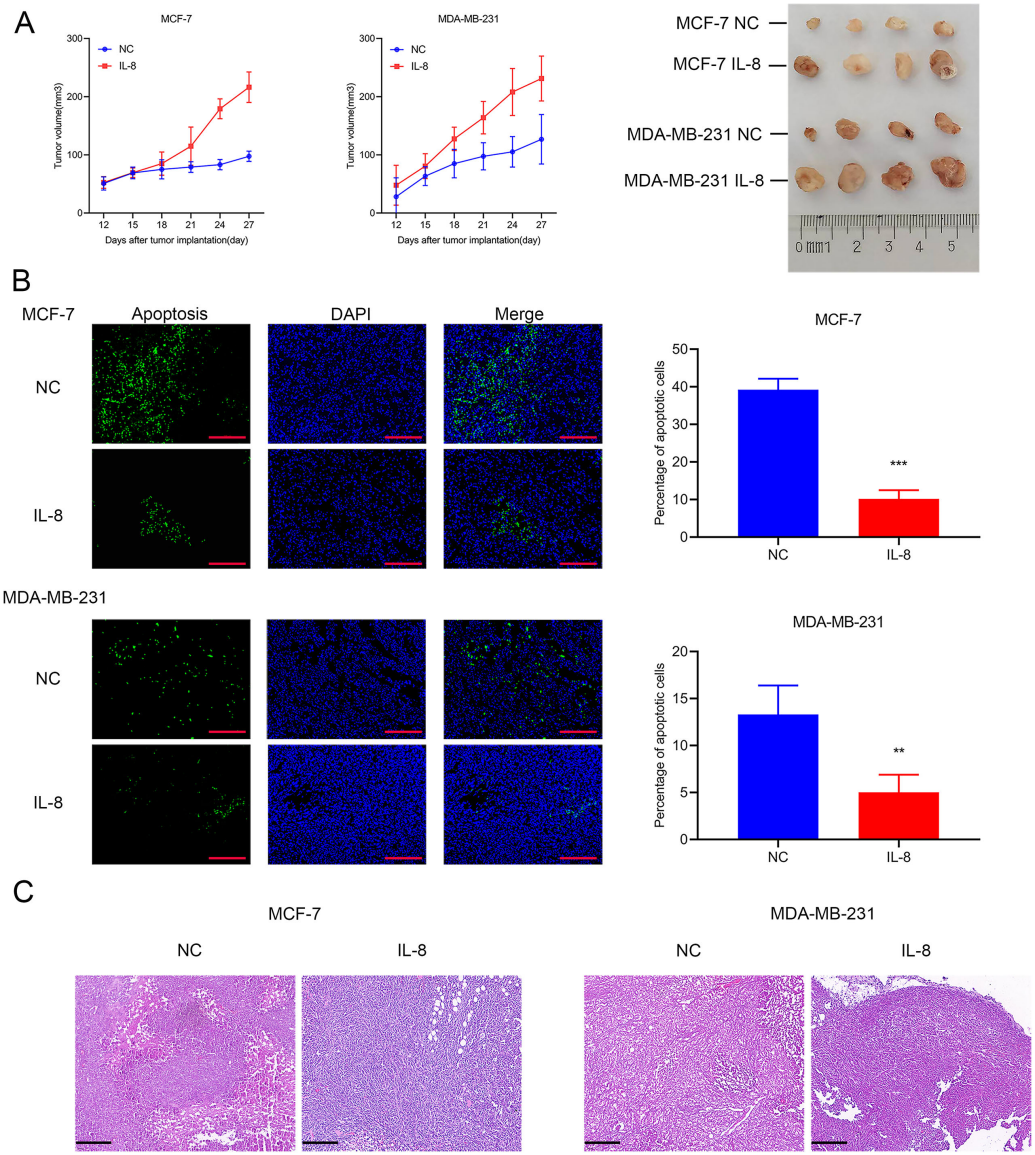
The effect of IL-8 on tumor growth *in vivo*. **(A)** Effect of IL-8 on the growth of MCF-7 and MDA-MB-231 xenografts *in vivo*. **(B)** TUNEL assay showed the effect of IL-8 on apoptosis in MCF-7 and MDA-MB-231 xenografts *in vivo*. Scale bar, 200 µm. Right panel, Quantitative analysis of TUNEL for apoptosis. **(C)** H&E staining of tumor tissues from MCF-7 and MDA-MB-231 xenografts. Scale bar, 50 µm. *P < 0.05, **P < 0.01, ***P < 0.001.

Furthermore, the xenograft model showed that IL-8 promoted tumor growth and reduced tumor cell apoptosis (**Figures 8A, B**). Importantly, H&E staining showed that the tumor tissues injected with IL-8 were more malignant, characterized by deeper nuclear staining, increased cellular atypia, denser cell arrangement, and fewer apoptotic tissues (**Figure 8C**). Taken together, these results suggested that IL-8 may play a tumor-promoting role in the progression of BRCA.

### 3.8 The mechanism of IL-8 mediates chemoresistance

**Figure 9:**
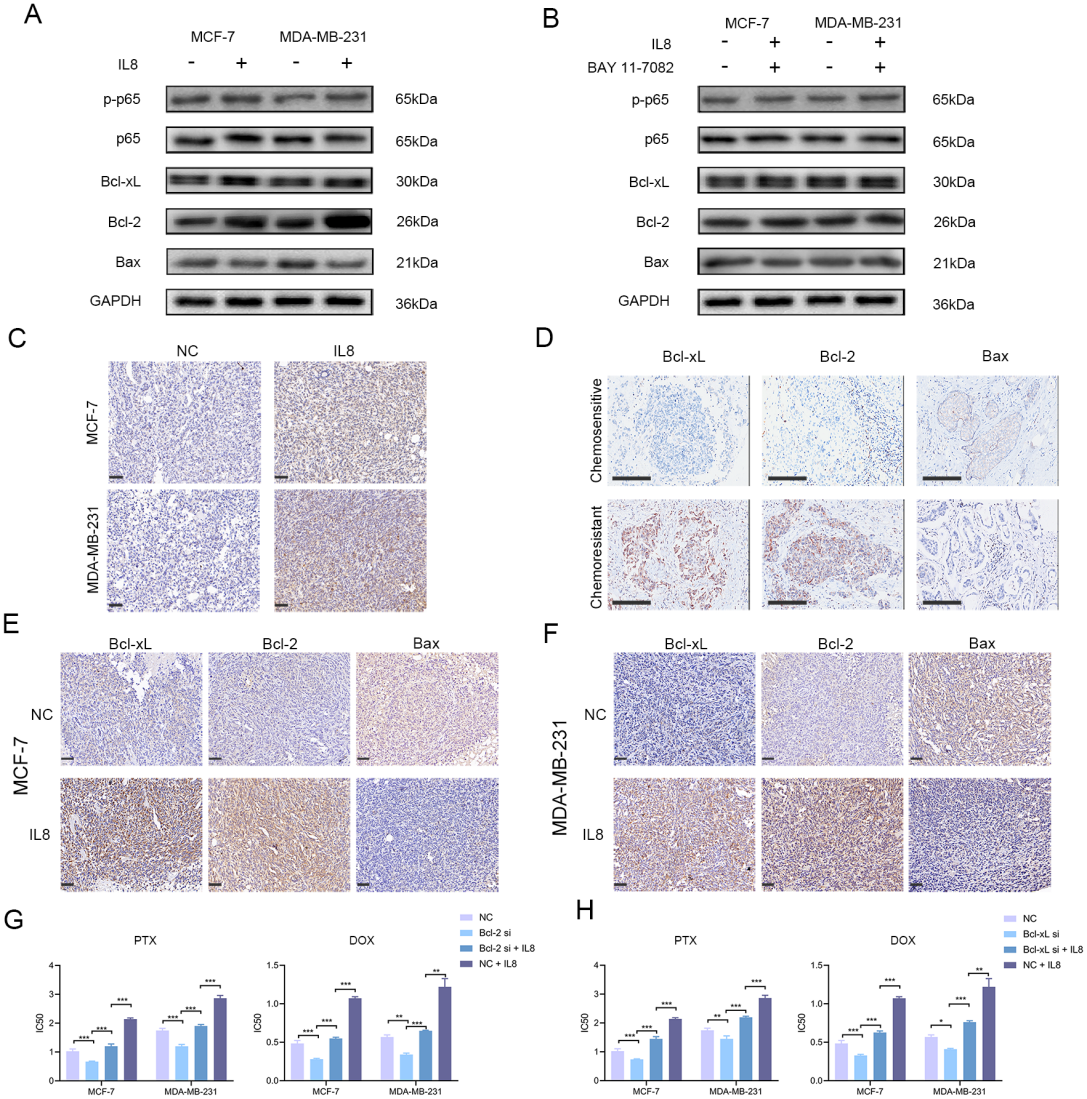
Mechanism of IL-8 induced chemoresistance. **(A, B)** Western blotting detected the expression of p-p65, Bcl-xL, Bcl-2, and Bax. **(C)** The expression of p-p65 in MCF-7 and MDA-MB-231 xenografts. Scale bar, 20 µm. **(D)** Compared with the chemosensitive group, Bcl-xL and Bcl-2 were overexpressed and Bax was weakly expressed in tumor tissues from chemoresistant group. Scale bar, 200 µm. **(E, F)** IL-8 promoted the expression of Bcl-xL and Bcl-2 and reduced Bax expression in MCF-7 and MDA-MB-231 xenografts. Scale bar, 20 µm. **(G, H)** The chemoresistance mediated by IL-8 was significantly attenuated upon Bcl-xL or Bcl-2 was knocked down in tumor cells. Data were shown as means ± SD for three independent experiments. *P < 0.05, **P < 0.01, ***P < 0.001.

We next sought to explore the underlying mechanisms associated with IL-8-mediated chemoresistance. It has been reported that IL-8 is associated with apoptosis, so we detected the expression of anti-apoptotic proteins Bcl-xL and Bcl-2, and pro-apoptotic protein Bax (27). We found that p-p65 expression was elevated in tumor cells after treatment with IL-8 (100 ng/ml) (**Figure 9A**), indicating that IL-8 promoted chemoresistance potentially through NF-κB pathway activation. At the same time, the expression of Bcl-xL and Bcl-2 was correspondingly increased, while Bax expression was decreased. Importantly, the addition of BAY 11-7082 (NF-κB pathway inhibitor) partially reversed the IL-8-mediated upregulation of Bcl-xL and Bcl-2, downregulation of Bax, and phosphorylation of p65 (**Figure 9B**). Furthermore, the IHC experiment revealed a significant upregulation of p-p65 expression in tumor xenograft tissues after IL-8 injection (**Figure 9C**). This observation further strengthens the link between IL-8 and NF-κB pathway activation. To further confirm that Bcl-xL, Bcl-2, and Bax are involved in chemoresistance, we performed IHC staining on tumor tissues from patients post-NAC. Notably, high expressions of Bcl-xL and Bcl-2, and low expression of Bax, were observed in tumor tissues from chemoresistant patients as compared to chemosensitive patients (**Figure 9D**). Similar results were observed in the tumor xenograft tissue (**Figures 9E, F**). To further validate the role of Bcl-xL and Bcl-2 in IL-8-mediated chemoresistance, we added IL-8 (100 ng/ml) to tumor cells after knocking down Bcl-xL or Bcl-2. We found that the chemoresistance mediated by IL-8 was significantly attenuated upon knockdown of Bcl-xL or Bcl-2 (**Figures 9G, H**). Therefore, we conclude that IL-8 upregulates the expression of anti-apoptotic proteins Bcl-xL and Bcl-2 and downregulates pro-apoptotic protein Bax expression by activating the NF-κB pathway, thereby leading to chemoresistance and poor prognosis.

### 3.9 IL-8 associated with chemoresistance and poor prognosis in BRCA patients

**Figure 10:**
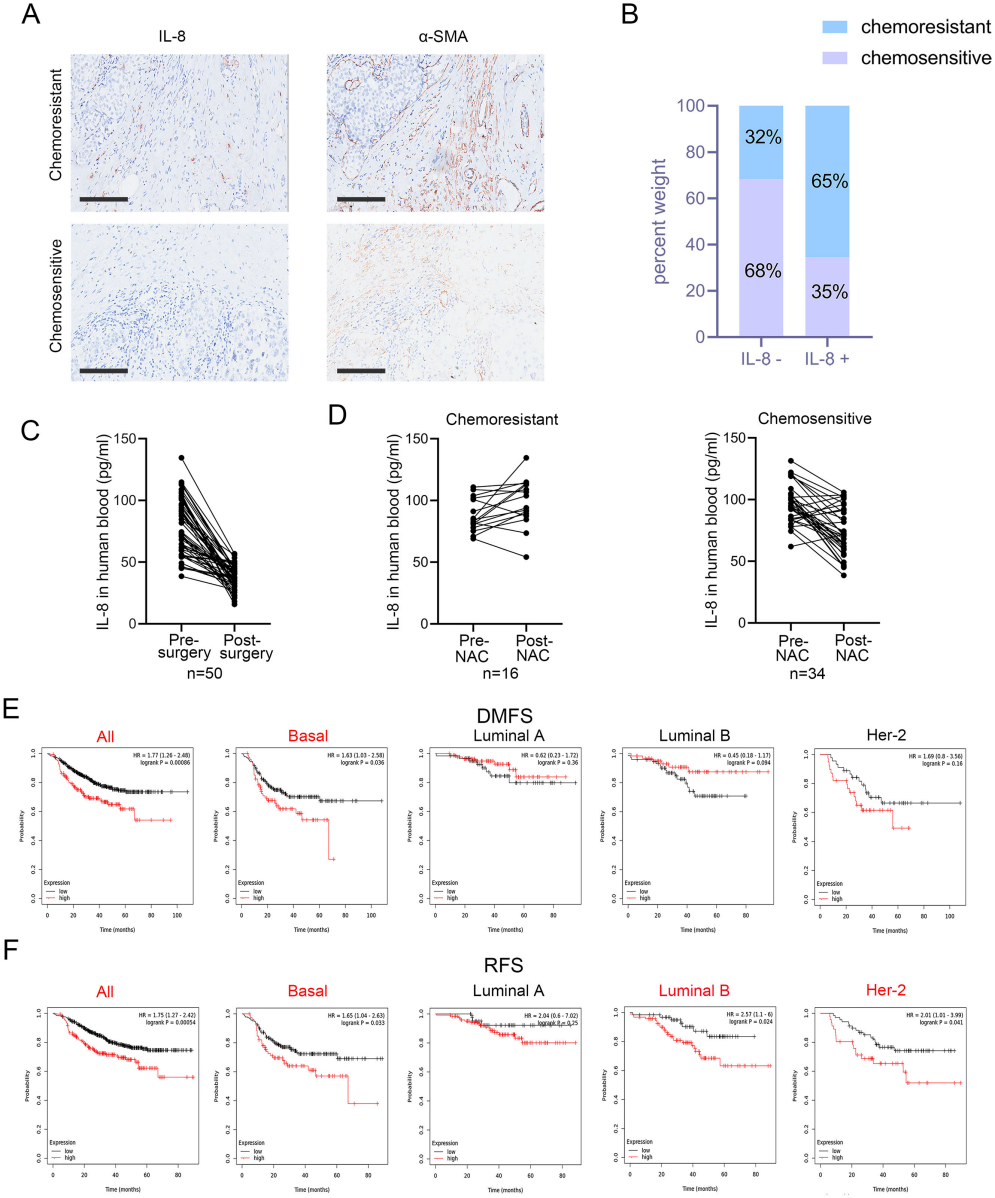
The expression signature and clinical prognosis of IL-8 in BRCA. **(A)** IL-8 was remarkably expressed in the tumor stroma of chemoresistant group, while it was barely expressed or lowly expressed in the stromal tissues of chemosensitive group. Scale bar, 200 µm. **(B)** Relationships between IL-8 expression and chemosensitivity (P < 0.001). **(C)** The serum IL-8 levels of all the patients decreased sharply after surgery (P < 0.001). **(D)** The serum IL-8 levels were significantly elevated in chemoresistant group (P < 0.05) but reduced in chemosensitive group (P < 0.001). **(E, F)** The prognostic value of IL-8 for DMFS and RFS in different subtypes of BRCA patients on NAC.

To explore the clinical significance of IL-8, we analyzed the clinicopathological characteristics of 100 BRCA patients who underwent NAC. We found that IL-8 expression was correlated with chemosensitivity and pTNM stage (**Table 3**). Importantly, we found that IL-8 was remarkably expressed in the tumor stroma of chemoresistant patients as compared to the chemosensitive patients (**Figures 10A, B**). To further assess whether IL-8 was associated with NAC efficacy, we collected serum from patients who received PTX- or DOX-based NAC. The serum IL-8 levels of all the patients decreased sharply after surgery (**Figure 10C**). It was obvious that serum IL-8 levels were significantly elevated in chemoresistant patients, while serum IL-8 levels in chemosensitive patients tended to be lower (**Figure 10D**). In conclusion, we demonstrated that IL-8 was associated with chemosensitivity in BRCA patients. Furthermore, data from the Kaplan-Meier Plotter showed that high IL-8 expression was associated with low distant metastasis-free survival (DMFS) and relapse free survival (RFS) in BRCA patients who received NAC (**Figures 10E, F**).

**Table 3:** IL-8 expression levels correlate with the clinicopathological characteristics of BRCA NAC patients.

Characteristics	N	IL-8 expression (tumor tissue)		P value
		Negative (n)	Positive (n)	
NAC				
Pre-NAC	100	96	4	
Post-NAC	100	74	26	< 0.001
Her-2				
–	45	29	16	
+	55	45	10	0.049
Age (years)				
< 60	56	45	11	
≥60	44	29	15	0.102
Lymph node metastasis				
N0/N1	63	48	15	
N2/N3	37	26	11	0.515
pTNM stage				
I II	37 43	22 37	15 6	
III	20	15	5	0.026
Chemotherapy				
Sensitive	61	52	9	
Resistant	39	22	17	0.001
Menopause				
N0	50	40	10	
Yes	50	34	16	0.171
Ki67(post-NAC)				
< 50	68	49	19	
≥50	32	25	7	0.519

## 4 Discussion

We present a comprehensive multi-omics and experimental investigation revealing that chemotherapy-induced IL-8 secretion from CAFs promotes tumor progression and undermines therapeutic efficacy. This finding aligns with the growing recognition that the TME plays a critical role in therapeutic response. We observed that both PTX and DOX significantly upregulated IL-8 expression in CAFs *in vitro* and *in vivo*. Critically, high levels of IL-8 were consistently associated with poor prognosis and chemoresistance across our clinical cohorts, MR analysis, and single-cell sequencing data. This suggests that the adaptive response of CAFs to chemotherapy is a key driver of treatment failure.

In addition to tumor cells, IL-8 can be secreted by fibroblasts, macrophages, and neutrophils (28). Several studies have shown that IL-8 in the TME exerts a pro-angiogenic function (29), and promotes drug resistance and metastasis of tumor cells by enhancing epithelial-mesenchymal transition (30), which is associated with a variety of malignant processes and adverse outcomes (31–33). In a large retrospective study, high baseline levels of IL-8 in patient serum was shown to be correlated with poor prognosis in advanced cancer patients (34). Extending these clinical observations, our MR analysis supported a causal association between genetically predicted IL-8 levels and BRCA risk, while scPagwas and BayesPrism deconvolution further identified IL-8 + CAFs as a key subpopulation linked to adverse outcomes in BRCA.

**Figure 11:**
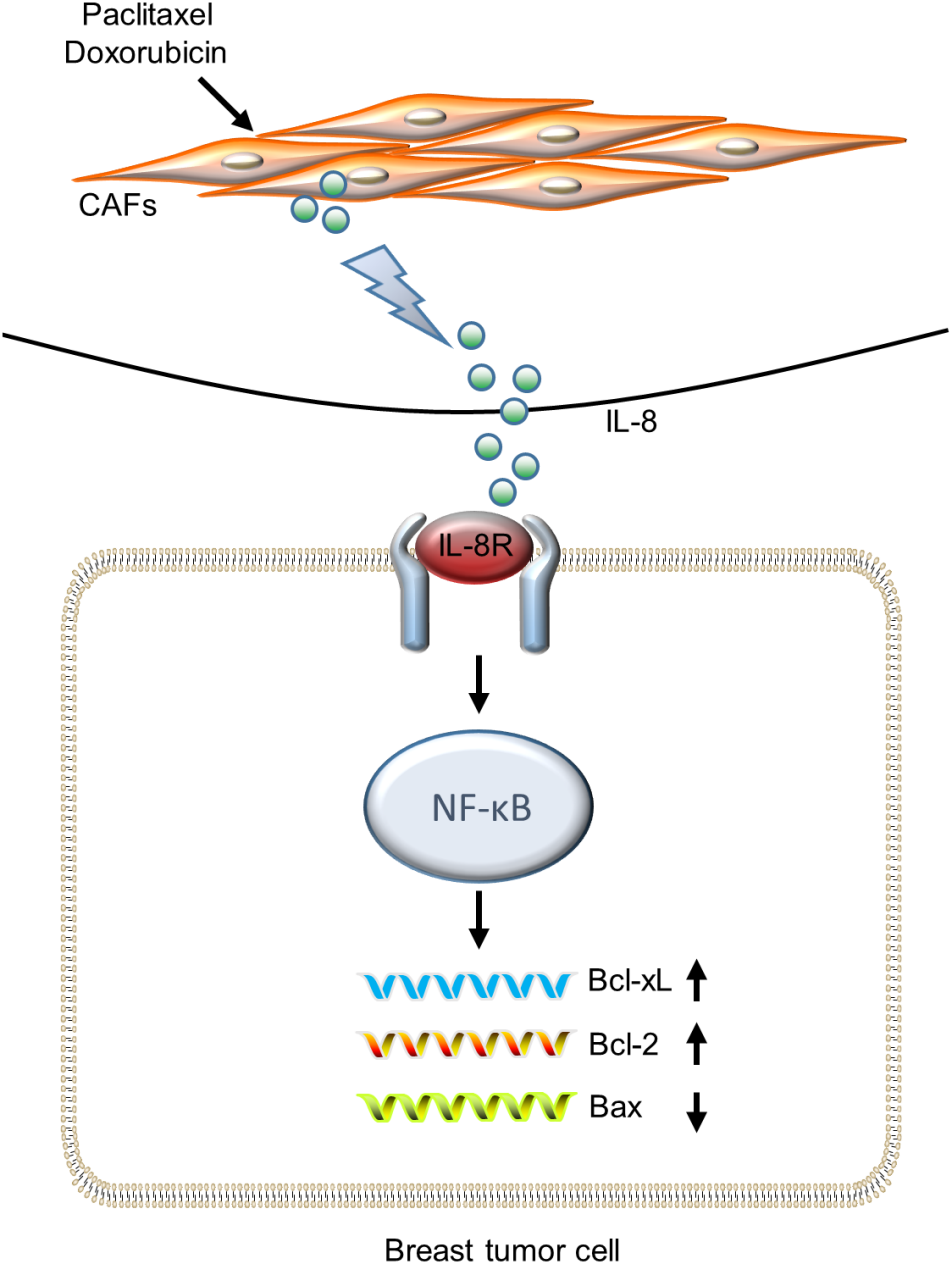
Schematic mechanism by which chemotherapy-treated CAFs promote chemoresistance.

To more closely replicate the TME in the patient, we employed the 3D micro-tumor PTCs model, and as expected, found IL-8 to reduce the sensitivity of PTCs to PTX or DOX. Meanwhile, data from the TUNEL apoptosis assay showed that IL-8 reduced PTX- or DOX-induced apoptosis, suggesting that the IL-8-induced reduction of chemosensitivity of tumor cells might be related to the suppression of apoptosis. Previously, another study showed that IL-8-induced chemoresistance was associated with increased expression of Bcl-xL and Bcl-2 (35). Bcl-xL and Bcl-2 are known to regulate cancer chemosensitivity, and numerous studies have reported that Bcl-xL and Bcl-2 are overexpressed in BRCA, prostate cancer, melanoma, and other human cancers (36–38). This overexpression protects tumor cells from chemotherapy-induced apoptosis and promotes a multidrug-resistant phenotype in tumor cells (39, 40). Most antitumor drugs promote cell death through apoptosis induction. The overexpression of anti-apoptotic proteins and the increased ratio of Bcl-2/Bax will reduce the chemosensitivity of tumor cells (41, 42). Here, we found that IL-8 upregulated the expression of Bcl-xL and Bcl-2 and downregulated Bax in BRCA cell lines and confirmed that Bcl-xL and Bcl-2 were overexpressed and Bax was weakly expressed in tumor tissues of chemoresistant BRCA patients. Therefore, we suggest that chemotherapy-induced IL-8 expression in CAFs upregulates the expression of Bcl-xL and Bcl-2 and downregulates Bax expression, by activating the NF-κB signaling pathway, which leads to chemoresistance in BRCA (**Figure 11**).

IL-8 functions as a pivotal signaling hub rather than a mere inflammatory marker. Upon binding to its cognate receptors CXCR1/2 on tumor cells, it triggers a robust phosphorylation cascade involving PI3K/Akt and NF-κB. This activation not only upregulates anti-apoptotic proteins to counteract chemotherapy-induced cytotoxicity, but also promotes epithelial-mesenchymal transition, thereby facilitating invasion and metastasis. Given the critical role of IL-8 in mediating chemoresistance, targeting this cytokine represents a promising therapeutic strategy. Recent evidence has highlighted the potential of anti-IL-8 monoclonal antibodies in BRCA treatment. Specifically, blocking IL-8 signaling has been shown to inhibit autophagic activity and impair the maintenance of cancer stem cells within the TME (43). Our findings further support this translational direction, suggesting that combining standard chemotherapy with IL-8 blockade could potentially dismantle the chemotherapy-induced protective stromal niche and sensitize tumor cells to cytotoxic agents.

Of course, our study has limitations. Primarily, we focused on IL-8 expression in CAFs, despite its presence in macrophages and other cells. Secondly, the expression of various cytokines in CAFs changes after chemotherapy, and IL-8 may play only a partial role. Therefore, whether the mechanism underlying CAFs-mediated progression is related to other cytokines warrants further exploration. Thirdly, our xenograft model used exogenous IL-8 injection rather than co-injection with chemotherapy-pretreated CAFs, which does not fully reflect the complex TME crosstalk. Future studies employing CAFs-tumor co-injection systems, particularly with patient-derived CAFs pre-exposed to PTX or DOX, are urgently needed to recapitulate the physiologically relevant context.

## 5 Conclusion

In summary, while chemotherapy reduces tumor size, it simultaneously appears to induce IL-8 expression in CAFs, driving tumors toward therapeutic resistance and metastasis. Our study highlights the potential counter-effects of chemotherapy-induced IL-8 secretion in CAFs on the efficacy of chemotherapy and suggests that IL-8 may be a potential target for cancer therapy. Conclusively, our findings provide insights into a potential mechanism of chemotherapy-induced chemoresistance, suggesting that targeting IL-8 may enhance chemotherapy efficacy.

## Data Availability

The original contributions presented in the study are included in the article/**Supplementary Material**, further inquiries can be directed to the corresponding author/s.

## Supplementary material

Supplementary Figure 1 **(A-D)** The processing and quality control of the single-cell data.

Supplementary Figure 2 **(A-C)** The transfection efficiency was detected by Elisa and Western blotting.
