# Correction: Integrating multi-omics data reveals IL-8 positive cancer-associated fibroblasts as mediators of chemotherapy-induced tumor progression in breast cancer

**DOI:** 10.3389/fimmu.2026.1947131

**Published:** 2026-08-03

**Authors:** Huifeng Liao, Huayan Li, Jin Song, Junhua Dong, Xue Bai

**Affiliations:** 1 Department of General Surgery, The First Medical Center of Chinese People’s Liberation Army General Hospital, Beijing, China; 2 The Second School of Clinical Medicine, Southern Medical University, Guangzhou, China; 3 Department of General Surgery, Seventh Medical Center of Chinese People’s Liberation Army General Hospital, Beijing, China; 4 Department of Breast Surgery, Fengtai Maternal and Child Health Hospital, Beijing, China

**Keywords:** cancer-associated fibroblasts, IL-8, Mendelian randomization, patient-derived tumor-like cell clusters, tumor microenvironment

There were some issues with the assignment of the affiliations to the authors in the published article. Below is the correct way the affiliations had to be assigned:

“Huifeng Liao^1,2,3^, Huayan Li^2^, Jin Song^4^, Junhua Dong^3^, Xue Bai^1,2^*”

“^1^Department of General Surgery, The First Medical Center of Chinese People’s Liberation Army General Hospital, Beijing, China

^2^The Second School of Clinical Medicine, Southern Medical University, Guangzhou, China

^3^Department of General Surgery, Seventh Medical Center of Chinese People’s Liberation Army General Hospital, Beijing, China

^4^Department of Breast Surgery, Fengtai Maternal and Child Health Hospital, Beijing, China”

The original version of this article has been updated.

