# Supplementary figures and images for: Integrating multi-omics data reveals IL-8 positive cancer-associated fibroblasts as mediators of chemotherapy-induced tumor progression in breast cancer
Source: Front Immunol. 2026 Jul 20;17:1878482. doi: 10.3389/fimmu.2026.1878482 (PMC13429397; doi:10.3389/fimmu.2026.1878482)

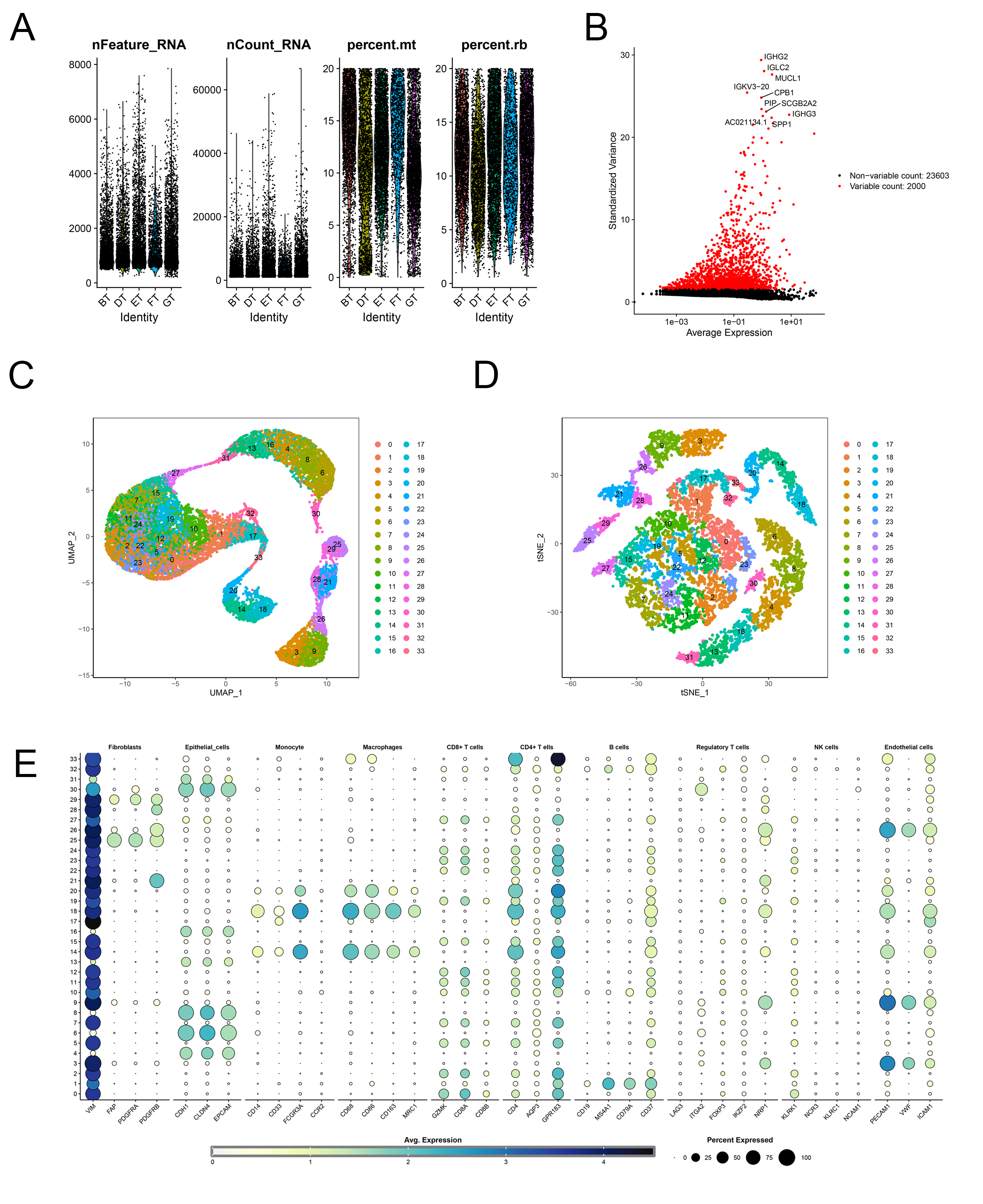

Supplement: Supplementary Figure 1 — (A-D) The processing and quality control of the single-cell data. [file Image1.tif]

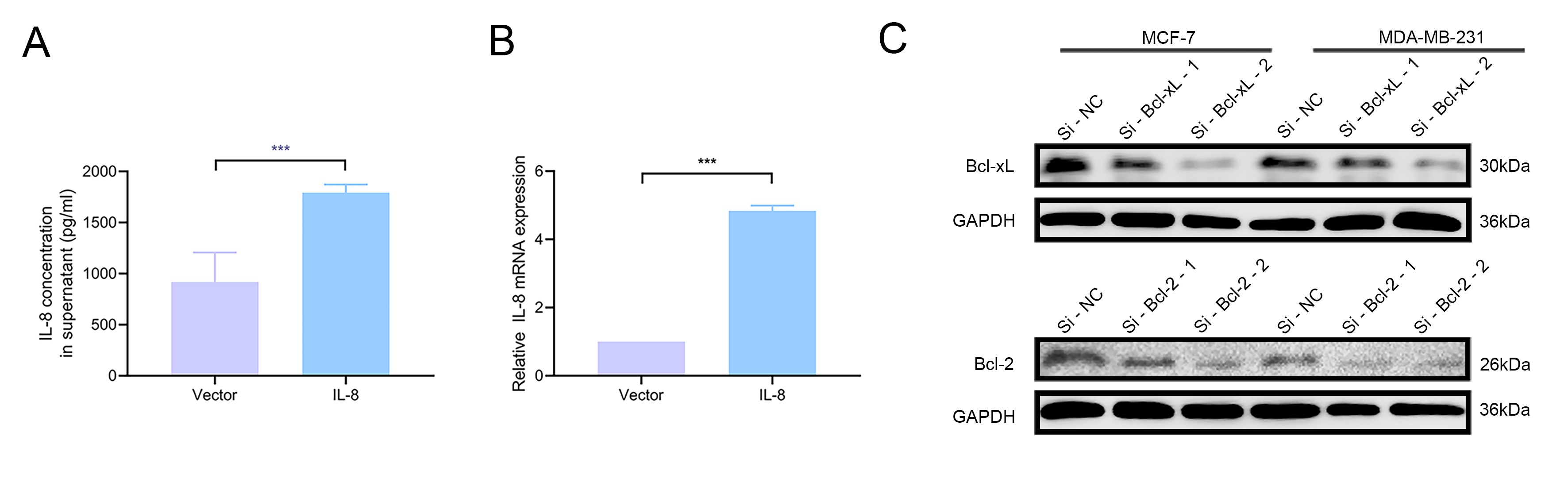

Supplement: Supplementary Figure 2 — (A-C) The transfection efficiency was detected by Elisa and Western blotting. [file Image2.tif]
